# Food Coma: Hyperammonemic Encephalopathy From Refeeding Syndrome

**DOI:** 10.7759/cureus.18898

**Published:** 2021-10-19

**Authors:** Joseph Khoory, Arashdeep Rupal, Chinmay Jani, Harpreet Singh, Kurt Hu

**Affiliations:** 1 Internal Medicine, Mount Auburn Hospital, Harvard Medical School, Cambridge, USA; 2 Critical Care Medicine, Medical College of Wisconsin, Milwaukee, USA; 3 Pulmonary and Critical Care Medicine, Medical College of Wisconsin, Milwaukee, USA

**Keywords:** hyperammonemia, encephalopathy, squamous cell carcinoma, refeeding syndrome, urea cycle disorder

## Abstract

Hyperammonemic encephalopathy (HAE) from extrahepatic causes is increasingly being recognized. Refeeding syndrome is characterized by severe fluid and electrolyte shifts following the reintroduction of nutrition. We describe the case of a 67-year-old man with bilateral maxillary sinus squamous cell carcinoma on nivolumab who became comatose after initiation of enteral feeding. Initial workup was notable for severe hypophosphatemia (<1 mg/dL) and markedly elevated ammonia (226 µmol/L). Neuroimaging was unrevealing. Correction of hypophosphatemia did not improve mental status. Ammonia levels briefly decreased while holding enteral feeding but worsened again on resumption. High-volume continuous renal replacement therapy was recommended but deferred in accordance with family wishes. We hypothesize that HAE may have been precipitated by a combination of refeeding-induced high nitrogen burden and limited detoxification via the urea cycle and extrahepatic pathways in the setting of severe protein-energy malnutrition and underlying malignancy. Nivolumab could have contributed as well.

## Introduction

Hyperammonemia is a metabolic disorder characterized by the elevation of ammonia in plasma, usually seen in decompensated liver cirrhosis. Extrahepatic causes are increasingly recognized in patients with hemato-oncological disorders, organ transplantation, and urease-producing bacterial infections, and portend a high mortality risk due to the acuity and severity of onset [1]. Refeeding syndrome (RFS) is a cascade of metabolic disturbances with an unclear incidence that results from severe fluid and electrolyte shifts following the reintroduction of nutrition in malnourished individuals [2]. Although many clinical scenarios can predispose patients to develop RFS, cancer patients appear to be at increased risk [3,4]. Here, we describe a case of rapid, severe encephalopathy in the setting of hypophosphatemia and hyperammonemia in a non-cirrhotic, malnourished cancer patient who was started on enteral nutrition, potentially suggesting a new non-hepatic etiology for acute hyperammonemic encephalopathy (HAE).

## Case presentation

A 67-year-old man with bilateral maxillary sinus squamous cell carcinoma was hospitalized for percutaneous gastrostomy tube (PEG) placement due to six months of inadequate oral intake secondary to oronasal fistula and consequent protein-energy malnutrition. He was diagnosed three years prior and had undergone multiple resections, including right radical maxillectomy, orbital exenteration, and left infratemporal fossa mass excision one year ago. Following surgical resection, he was treated with nivolumab (Winship trial) and concurrent radiation therapy; however, his treatment course was complicated by posterior dehiscence of the soft palate leading to the development of a sizable oronasal fistula, significantly limiting oral intake. Before the development of the oronasal fistula, he was able to eat and drink a texture-modified pureed diet in addition to nutrition supplementation (BOOST Plus®, Nestle HealthCare, Fremont, MI) one to three times per day. Severe protein-energy malnutrition was evident from loss of body fat, significant muscle wasting, >50% decreased oral intake, and >7.5% weight loss in the preceding three months. Other medical history included hypertension, hypothyroidism, and peripheral artery disease. His medications included aspirin, atorvastatin, metoprolol, gabapentin, levothyroxine, and vitamin supplements including thiamine. Enteral tube feeding with Osmolite 1.5 (OSMOLITE®, Abbott Nutritional Products, Abbott Park, IL) was initiated on day three of hospitalization (12 hours post-PEG tube placement) at 10 Kcal/kg/day along with 90 g daily protein. Forty-eight hours after initiation of enteral nutrition, he became unarousable (Glasgow Coma Scale score of 5). Initial workup was notable for a significant decrease in phosphate level to <1 mg/dL, from the presenting level of 3.4 mg/dL, and markedly increased venous ammonia (226 µmol/L); of note, no baseline ammonia level was obtained. Hypophosphatemia was promptly corrected (3.1 mg/dL), and enteral tube feeding was held. Offending medications, which only included gabapentin, were held. Despite this, his comatose state did not improve. Computed tomography (CT) scans of the head and abdomen were unremarkable, and an electroencephalogram showed only moderate-to-severe background slowing. Sodium phenylacetate (nitrogen scavenger), lactulose, and rifaximin were started. Ammonia levels improved (161 µmol/L) 24 hours after intervention, but worsened to 177 µmol/L within 12 hours of resumption of his enteral feeding, even at a lower caloric count (Osmolite 1.5; 1.5 Kcal) and protein load. High-volume continuous renal replacement therapy (CRRT) was recommended because of the persistent comatose state and suspected intracranial hypertension but deferred in accordance with family wishes. He was subsequently transitioned to hospice and passed away.

## Discussion

Encephalopathy during RFS may be caused by profound hypophosphatemia, thiamine deficiency, and central pontine myelinolysis [5,6]. Within days, malnourished patients gradually develop total body depletion of vitamins and minerals, particularly phosphorus, due to the transition from carbohydrate to fat metabolism, falling insulin levels, and reversing the anabolic state [7]. In conjunction with total body potassium and magnesium depletion, RFS presents with multisystem abnormalities and is a nutritional and metabolic emergency. In our patient, thiamine was supplemented prophylactically before initiating enteral tube feeding to prevent Wernicke’s encephalopathy [2]. Neuroimaging excluded acute neurological catastrophes. Prompt evaluation of his encephalopathy did not reveal anything specific, except for severe hyperammonemia with an unclear etiology.

The predominant cause of non-congenital hyperammonemia is impaired clearance in cirrhosis. However, non-hepatic causes are increasingly recognized [1,8]. These include hemato-oncological disorders, urease-producing infections, unmasked urea cycle defects, and medications. Additionally, medical conditions predisposing to nutritional complications such as gastric bypass and high-protein diets have also been implicated in involving urea cycle disruptions [9,10]. In rats, zinc deficiency has been shown to decrease ornithine transcarbamylase activity, a key enzyme in the urea cycle [11]. Conditions associated with severe phosphate depletion, such as in our case with RFS, have been theorized as a cause of hyperammonemia, but no established link has been demonstrated [12].

HAE has been reported in the case of hepatocellular carcinoma. Proposed hypotheses include increased cellular breakdown due to chemotherapy, intrahepatic shunting of ammonia, and a paraneoplastic process that disrupts the urea cycle decreasing ammonia clearance [13]. Our patient had no evidence of a primary urea cycle defect or an underlying liver disease. He had a history of maxillary sinus squamous cell carcinoma and was being treated with nivolumab and concurrent radiation therapy. This therapy could have caused an increased cellular breakdown resulting in large quantities of additional nitrogenic waste. Nivolumab has also been associated with autoimmune hepatitis, leading to HAE, although our patient had normal liver function tests. Tumor lysis syndrome was considered, but laboratory results were inconsistent [14].

A similar case was reported in which a 59-year-old woman with Crohn’s disease with no preexisting liver disease developed refeeding encephalopathy with severe hypophosphatemia and hyperammonemia. Following normalization of phosphate level, she underwent hemodialysis to lower her ammonia level resulting in the improvement in her mental status [15]. Unfortunately, in our case, correction of hypophosphatemia alone did not improve the comatose state, and the patient’s family declined CRRT.

Cancer patients and individuals with head and neck cancer are especially vulnerable to developing RFS [16]. In an observational study of 54 patients with head and neck cancer, 11 (20%) developed RFS. The risk factors most strongly associated were pain, eating difficulties, high alcohol intake, low handgrip strength, and previous radiation therapy [17]. Underlying metabolic and electrolyte derangements due to cancer also contribute [16,18].

Universal recommendations for safely advancing nutrition are not feasible due to heterogeneity in the patient population. Studies have generally favored a cautious approach for critically ill patients versus a more aggressive regimen for patients with anorexia nervosa [17]. General guidelines combine careful reinitiation with close monitoring of electrolytes and fluid status [19]. Even though there is a paucity of evidence regarding protein restriction, reducing nitrogen burden is crucial [17,20,21]. Urgent treatment includes high-volume CRRT while reversing the underlying cause; nitrogen scavengers have also been used in refractory cases [1]. High-volume CRRT is considered superior to intermittent hemodialysis because of fewer chances of rebound hyperammonemia and complications from rapid shifts in osmolarity [22]. Comprehensive care incorporating nutritionists is essential in all cancer patients and especially those at risk before initiating nutrition.

## Conclusions

Identification of patients at a high risk of RFS is important. In our case, we hypothesize that HAE was caused by a combination of refeeding-induced high nitrogen burden and limited detoxification via the urea cycle and extrahepatic pathways in the setting of severe protein-energy malnutrition due to the underlying cancer state.

## References

[1] Stergachis AB, Mogensen KM, Khoury CC (2020). A retrospective study of adult patients with noncirrhotic hyperammonemia. J Inherit Metab Dis.

[2] Crook MA, Hally V, Panteli JV (2001). The importance of the refeeding syndrome. Nutrition.

[3] Marinella MA (2009). Refeeding syndrome: an important aspect of supportive oncology. J Support Oncol.

[4] Stanga Z, Brunner A, Leuenberger M, Grimble RF, Shenkin A, Allison SP, Lobo DN (2008). Nutrition in clinical practice-the refeeding syndrome: illustrative cases and guidelines for prevention and treatment. Eur J Clin Nutr.

[5] Mégarbane B, Guerrier G, Blancher A, Meas T, Guillausseau PJ, Baud FJ (2007). A possible hypophosphatemia-induced, life-threatening encephalopathy in diabetic ketoacidosis: a case report. Am J Med Sci.

[6] Ardalan MR, Pourafkari L, Tubbs RS, Shoja MM (2008). Hypophosphatemic encephalopathy in a CAPD patient. Am J Med Sci.

[7] Walmsley RS (2013). Refeeding syndrome: screening, incidence, and treatment during parenteral nutrition. J Gastroenterol Hepatol.

[8] Sakusic A, Sabov M, McCambridge AJ (2018). Features of adult hyperammonemia not due to liver failure in the ICU. Crit Care Med.

[9] Estrella J, Yee G, Wilcken B, Tchan M, Talbot M (2014). Hyperammonemic encephalopathy complicating bariatric surgery: a case study and review of the literature. Surg Obes Relat Dis.

[10] Singh S, Suresh S, McClave SA, Cave M (2015). Treating every needle in the haystack: hyperammonemic encephalopathy and severe malnutrition after bariatric surgery-a case report and review of the literature. JPEN J Parenter Enteral Nutr.

[11] Riggio O, Merli M, Capocaccia L (1992). Zinc supplementation reduces blood ammonia and increases liver ornithine transcarbamylase activity in experimental cirrhosis. Hepatology.

[12] Becker S, Dam G, Hvas CL (2015). Refeeding encephalopathy in a patient with severe hypophosphataemia and hyperammonaemia. Eur J Clin Nutr.

[13] Cho J, Chen JC, Paludo J, Conboy EE, Lanpher BC, Alberts SR, Halfdanarson TR (2019). Hyperammonemic encephalopathy in a patient with fibrolamellar hepatocellular carcinoma: case report and literature review. J Gastrointest Oncol.

[14] Yen TH, Chang CH, Shiu SI (2020). Tumor lysis syndrome after combination therapy of nivolumab and sorafenib in a woman with advanced hepatocellular carcinoma. Case Rep Gastroenterol.

[15] Lee JC, Lai HS, Huang SM, Chang CJ, Wang ST, Chen WJ (1994). Hyperammonemic encephalopathy due to essential amino acid hyperalimentation. J Formos Med Assoc.

[16] Windpessl M, Mayrbaeurl B, Baldinger C, Tiefenthaller G, Prischl FC, Wallner M, Thaler J (2017). Refeeding syndrome in oncology: report of four cases. World J Oncol.

[17] da Silva JS, Seres DS, Sabino K (2020). ASPEN consensus recommendations for refeeding syndrome. Nutr Clin Pract.

[18] Rasmussen SO, Kristensen MB, Wessel I, Andersen JR (2016). Incidence and risk factors of refeeding syndrome in head and neck cancer patients-an observational study. Nutr Cancer.

[19] Doig GS, Simpson F, Heighes PT (2015). Restricted versus continued standard caloric intake during the management of refeeding syndrome in critically ill adults: a randomised, parallel-group, multicentre, single-blind controlled trial. Lancet Respir Med.

[20] (2021). Nutrition support for adults: oral nutrition support, enteral tube feeding and parenteral nutrition. http://www.nice.org.uk/guidance/cg32.

[21] Häberle J, Boddaert N, Burlina A (2012). Suggested guidelines for the diagnosis and management of urea cycle disorders. Orphanet J Rare Dis.

[22] Raina R, Bedoyan JK, Lichter-Konecki U (2020). Consensus guidelines for management of hyperammonaemia in paediatric patients receiving continuous kidney replacement therapy. Nat Rev Nephrol.

